# Proteogenomics analysis of CUG codon translation in the human pathogen *Candida albicans*

**DOI:** 10.1186/s12915-021-01197-9

**Published:** 2021-12-04

**Authors:** Stefanie Mühlhausen, Hans Dieter Schmitt, Uwe Plessmann, Peter Mienkus, Pia Sternisek, Thorsten Perl, Michael Weig, Henning Urlaub, Oliver Bader, Martin Kollmar

**Affiliations:** 1 Theoretical Computer Science and Algorithmic Methods Group, Institute of Computer Science, University of Göttingen, Goldschmidtstr. 7, 37077 Göttingen, Germany; 2 Department of Neurobiology, Max-Planck-Institute for Biophysical Chemistry, Am Fassberg 11, 37077 Göttingen, Germany; 3 Bioanalytical Mass Spectrometry, Max-Planck-Institute for Biophysical Chemistry, Am Fassberg 11, 37077 Göttingen, Germany; 4 Institute for Medical Microbiology, University Medical Center Göttingen, Kreuzbergring 57, 37075 Göttingen, Germany; 5 Intermediate Care, University Medical Center Göttingen, Robert Koch Strasse 40, 37075 Göttingen, Germany; 6 Bioanalytics Group, Department of Clinical Chemistry, University Medical Center Göttingen, Robert Koch Strasse 40, 37075 Göttingen, Germany; 7 Group Systems Biology of Motor Proteins, Department of NMR-based Structural Biology, Max-Planck-Institute for Biophysical Chemistry, Am Fassberg 11, 37077 Göttingen, Germany

**Keywords:** Proteogenomics, Pathogen, *Candida albicans*, Genetic code

## Abstract

**Background:**

Yeasts of the CTG-clade lineage, which includes the human-infecting *Candida albicans*, *Candida parapsilosis* and *Candida tropicalis* species, are characterized by an altered genetic code. Instead of translating CUG codons as leucine, as happens in most eukaryotes, these yeasts, whose ancestors are thought to have lost the relevant leucine-tRNA gene, translate CUG codons as serine using a serine-tRNA with a mutated anticodon, ${\mathrm{tRNA}}_{\mathrm{CAG}}^{\mathrm{Ser}}$. Previously reported experiments have suggested that 3–5% of the CTG-clade CUG codons are mistranslated as leucine due to mischarging of the ${\mathrm{tRNA}}_{\mathrm{CAG}}^{\mathrm{Ser}}$. The mistranslation was suggested to result in variable surface proteins explaining fast host adaptation and pathogenicity.

**Results:**

In this study, we reassess this potential mistranslation by high-resolution mass spectrometry-based proteogenomics of multiple CTG-clade yeasts, including various *C. albicans* strains, isolated from colonized and from infected human body sites, and *C. albicans* grown in yeast and hyphal forms. Our data do not support a bias towards CUG codon mistranslation as leucine. Instead, our data suggest that (i) CUG codons are mistranslated at a frequency corresponding to the normal extent of ribosomal mistranslation with no preference for specific amino acids, (ii) CUG codons are as unambiguous (or ambiguous) as the related CUU leucine and UCC serine codons, (iii) tRNA anticodon loop variation across the CTG-clade yeasts does not result in any difference of the mistranslation level, and (iv) CUG codon unambiguity is independent of *C. albicans*’ strain pathogenicity or growth form.

**Conclusions:**

Our findings imply that *C. albicans* does not decode CUG ambiguously. This suggests that the proposed misleucylation of the ${\mathrm{tRNA}}_{\mathrm{CAG}}^{\mathrm{Ser}}$ might be as prevalent as every other misacylation or mistranslation event and, if at all, be just one of many reasons causing phenotypic diversity.

**Supplementary Information:**

The online version contains supplementary material available at 10.1186/s12915-021-01197-9.

## Background

*Candida* species belong to the most common fungal pathogens. About 90% of *Candida* infections are caused by just five *Candida* species: *Candida albicans*, *Candida parapsilosis*, *Candida tropicalis*, *Candida krusei* (synonyms *Issatchenkia orientalis*, or lately *Pichia kudriavzevii* [1]), and *Candida glabrata*. The first three of these belong to the so-called CTG-clade, which is termed as such because it only comprises species that have reassigned the CUG codon from leucine to serine [2]. However, CUG codon reassignment is not unique to CTG-clade species but is also observed in the *Ascoidea*-clade and in a lineage comprising *Pachysolen* and *Nakazawaea* yeasts [3–5]. While the CUG codon is translated as serine in most *Ascoidea*-clade species, *Ascoidea asiatica* exceptionally translates CUG stochastically into both serine and leucine [4]. In *Pachysolen* and *Nakazawaea* species CUG is translated into alanine [3, 4]. The CUG decoding ${\mathrm{tRNA}}_{\mathrm{CAG}}^{\mathrm{Ser}}$ in the CTG-clade originated from a ${\mathrm{tRNA}}_{\mathrm{HGA}}^{\mathrm{Ser}}$ isoacceptor (for UCU, UCG and UCA codons), whereas the ${\mathrm{tRNA}}_{\mathrm{CAG}}^{\mathrm{Ser}}$ in *Ascoidea*-species originated from a ${\mathrm{tRNA}}_{\mathrm{GCU}}^{\mathrm{Ser}}$ (for AGY codons) [4]. Accordingly, the long-favoured “ambiguous intermediate” model for CUG reassignment became extremely unlikely as it would require multiple independent events including divergence of multiple different tRNA types as ambiguous intermediates. Currently, the best model to explain these reassignments is the “tRNA-loss driven codon reassignment” hypothesis [3, 6]. According to this model, the ${\mathrm{tRNA}}_{\mathrm{CAG}}^{\mathrm{Leu}}$ was lost in the last common ancestor of Ascoideae, Pichiaceae, Saccharomycetaceae, and CTG-clade yeasts. The then unassigned CUG codon was captured by leucine-, serine-, and alanine-tRNAs which are incidentally the only tRNA types where anticodons are not part of tRNA identity elements [7].

The two CTG-clade species *C. albicans* and *Candida maltosa* have been reported to translate the reassigned CUG codon by 3–5% into leucine [8, 9]. Central to *C. albicans*’ pathogenicity is the ability to change the cellular morphology between the yeast and mycelial forms [10]. In this process, stochasticity of cell surface proteins might increase *C. albicans*’ ability to host adaptation [11]. In contrast, other species of the CTG-clade including *Candida cylindracea*, *Clavispora lusitaniae*, and *Babjeviella inositovora* have been reported to translate CUG unambiguously [4, 8]. Ambiguity of translation was proposed to depend on tRNA sequence. In particular, a guanosine at position 33, 5′-adjacent to the CAG anticodon (G33), and a 1-methyl guanosine nucleotide at position 37, 3′-adjacent to the anticodon (m^1^G37), were identified to be invariant in all ${\mathrm{tRNA}}_{\mathrm{CAG}}^{\mathrm{Ser}}$. The only exception at that time with only limited sequence data available was the *Candida cylindracea* tRNA in which an adenosine is found at position 37 (A37) [12, 13]. Mutating the G33 increased the affinity towards LeuRS (leucine-tRNA synthetase) thus enhancing leucylation [8]. Replacing the m^1^G37 by another nucleotide was shown to suppress leucylation [8]. This suggested a balancing effect of G33 and m^1^G37 as leucylation suppressor and enhancer, respectively, leading to low-level mischarging of CTG-clade ${\mathrm{tRNA}}_{\mathrm{CAG}}^{\mathrm{Ser}}$ with leucine [14]. Incorporation of 3–5% of leucine at CUG was reported from a genetic rescue experiment [8] and mass spectrometry of an overexpressed peptide [9]. In contrast, very low levels of mistranslation to no mistranslation by leucine at all (1.45 ± 0.85% in a *C. albicans* control and 0.64 ± 0.82% in a knock-out of one of the two ${\mathrm{tRNA}}_{\mathrm{CAG}}^{\mathrm{Ser}}$) were found in fluorescence measurements [15]. Similarly, we observed CUG translation into leucine only at background ribosomal mistranslation rates in high-resolution proteogenomics experiments in *Clavispora lusitaniae* and *Babjeviella inositovora* [4]. These results prompted us to reinvestigate CUG codon translation across the CTG-clade performing state-of-the-art proteogenomics analyses.

## Results

### Selection of yeasts for analysis

**Fig. 1.**
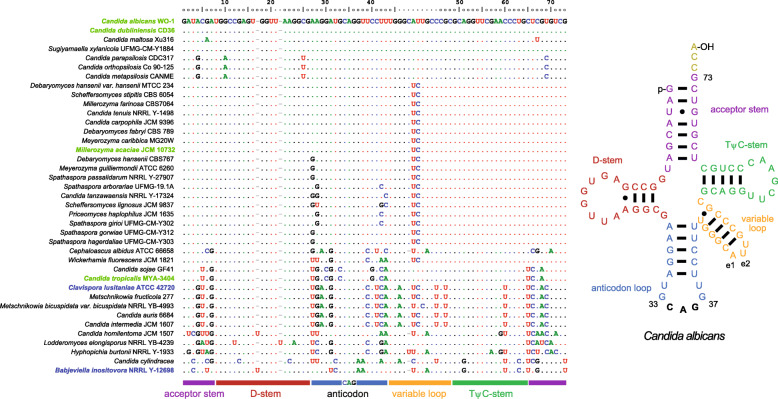
Serine-tRNA(CAG) diversity in CTG-clade yeasts. Alignment of ${\mathrm{tRNA}}_{\mathrm{CAG}}^{\mathrm{Ser}}$ from 38 sequenced yeast genomes and *Candida cylindracea*. Nucleotides identical to the *Candida albicans* ${\mathrm{tRNA}}_{\mathrm{CAG}}^{\mathrm{Ser}}$ are represented by dots to better highlight divergence. Numbering of nucleotide sequence positions is according to standard nuclear tRNA numbering [16]. Species with high-resolution proteomics data available from previous studies [4] highlighted in blue, species analysed in this study highlighted in green

To select the most appropriate species to determine the accuracy of CUG codon translation dependent on tRNA identity elements in vivo across the CTG-clade, we aligned ${\mathrm{tRNA}}_{\mathrm{CAG}}^{\mathrm{Ser}}$ from *C. cylindracea* and 38 sequenced species (Fig. 1) [4]. The ${\mathrm{tRNA}}_{\mathrm{CAG}}^{\mathrm{Ser}}$ most similar to that of *C. cylindracea* is *B. inositovora.* Both have an A37 instead of the common m^1^G37, a substitution which has been suggested to suppress mischarging by the LeuRS [13]. This might explain the observed unambiguous translation of CUG as serine in *B. inositovora* [4]. *C. lusitaniae*’s ${\mathrm{tRNA}}_{\mathrm{CAG}}^{\mathrm{Ser}}$ is as diverged from *C. albicans* ${\mathrm{tRNA}}_{\mathrm{CAG}}^{\mathrm{Ser}}$ as *B. inositovora* and *C. cylindracea* ${\mathrm{tRNA}}_{\mathrm{CAG}}^{\mathrm{Ser}}$ are from the *C. albicans* ${\mathrm{tRNA}}_{\mathrm{CAG}}^{\mathrm{Ser}}$, but the differences compared to the *C. albicans* ${\mathrm{tRNA}}_{\mathrm{CAG}}^{\mathrm{Ser}}$ are at non-overlapping positions (Fig. 1). *C. lusitaniae* was also shown to translate CUG unambiguously [4]. If *C. albicans* translated CUG ambiguously, as reported previously [8, 9], the reason could not be the anticodon loop, as this is identical between *C. albicans* and *C. lusitaniae* ${\mathrm{tRNA}}_{\mathrm{CAG}}^{\mathrm{Ser}}$ (Fig. 1). Instead, ambiguous translation would have to be due to a single or a combination of some of the 21 nucleotide differences spread across the other loops. To best represent ${\mathrm{tRNA}}_{\mathrm{CAG}}^{\mathrm{Ser}}$ diversity seen across the CTG-clade, we selected *Candida tropicalis*, *Millerozyma acaciae*, *Candida dubliniensis*, and *C. albicans* for an in-depth analysis. *C. tropicalis* ${\mathrm{tRNA}}_{\mathrm{CAG}}^{\mathrm{Ser}}$ and the identical *Candida sojae* ${\mathrm{tRNA}}_{\mathrm{CAG}}^{\mathrm{Ser}}$ are the only ${\mathrm{tRNA}}_{\mathrm{CAG}}^{\mathrm{Ser}}$ with a cytosine at position 33 (Fig. 1). Substitution of the conserved G33 by cytosine has been shown to strongly enhance leucylation activity in vitro [8]*. M. acaciae* ${\mathrm{tRNA}}_{\mathrm{CAG}}^{\mathrm{Ser}}$ differs from *C. albicans* ${\mathrm{tRNA}}_{\mathrm{CAG}}^{\mathrm{Ser}}$ only in ^e1^U^e2^C at the tip of the variable loop. This sequence is found in most CTG-clade yeast ${\mathrm{tRNA}}_{\mathrm{CAG}}^{\mathrm{Ser}}$ (Fig. 1). Corresponding nucleotides in *C. albicans*’ ${\mathrm{tRNA}}_{\mathrm{CAG}}^{\mathrm{Ser}}$ are ^e1^A^e2^U. To determine whether other factors than ${\mathrm{tRNA}}_{\mathrm{CAG}}^{\mathrm{Ser}}$ sequence could influence CUG translation ambiguity, we selected *C. dubliniensis*, whose ${\mathrm{tRNA}}_{\mathrm{CAG}}^{\mathrm{Ser}}$ is identical to that of *C. albicans*, and we choose multiple different *C. albicans* strains. In addition, we analysed *C. albicans* in yeast and hyphal growth forms. *C. albicans*, *C. dubliniensis* and *C. tropicalis* are diploids, the ploidy of *M. acacia* is, to our best knowledge, unknown.

CTG-clade ${\mathrm{tRNA}}_{\mathrm{CAG}}^{\mathrm{Ser}}$ are undoubtedly Ser-tRNAs [3, 4] but differ within the group in up to 25% of their nucleotides. They all have the Leu-tRNA CAG anticodon triplet and most have m^1^G37, which is present in most but not all Leu-tRNAs. Based on these few identities, it seems exaggerated to term the CTG-clade ${\mathrm{tRNA}}_{\mathrm{CAG}}^{\mathrm{Ser}}$ “chimaeric” tRNAs. A more concise use of “chimaera” would encompass only those entities (molecules, proteins, RNAs) consisting of clearly distinct parts of independent origins with each having a substantial impact on the entity. However, the ${\mathrm{tRNA}}_{\mathrm{CAG}}^{\mathrm{Ser}}$ originated from a ${\mathrm{tRNA}}_{\mathrm{HGA}}^{\mathrm{Ser}}$ isoacceptor followed by single-nucleotide insertion and/or point mutation and not by joining pieces from different tRNA genes [4].

### Unbiased peptide spectrum matching with database replicates

To determine the CUG codon translation in the selected yeasts, we performed liquid chromatography-tandem mass spectrometry (LC-MS/MS) analysis of cell lysates (Additional file 1). We did not employ a de novo peptide sequencing approach as this overstates the number of rare peptides [17, 18]. This approach mistakenly suggested 10% of CUGs translated by leucine in *Pachysolen tannophilus* [19] instead of the unambiguous translation (> 99%) into alanine as reported elsewhere [3]. In contrast, database searches with unbiased databases allow precise detection of rare peptides with a precision of single amino acid differences being mapped to identical protein regions, which can result from low-level ribosomal mistranslation [3] or stochastic translation as in *Ascoidea asiatica* [4]. Thus, we followed our previously described approach and generated an unbiased database for each analysed species containing each CUG codon (or, respectively, another codon of interest) translated into each of the 19 amino acids (leucine and isoleucine are indistinguishable by MS/MS and thus translation into isoleucine was omitted). To avoid impractical search times and loss of statistical power due to large sequence databases, we designed an approach to remove sequence redundancy as far as possible. Simulating an in silico trypsin digestion, all peptides containing CUG codon translations were fused with up to two neighbouring peptides at the N- and the C-terminus such that peptides with up to two missed cleavages can be found. All other peptides were fused as far as they belong to continuous stretches of the same protein and the 19 replicates were uniquified. By this approach, the search databases increased by only about 80%, compared to the gene prediction dataset using the standard genetic code. Our approach is an alternative to the approach that Mordret et al. used to resolve the far larger problem when searching for all possible mistranslations [20]. They used a stepwise approach: First, the data were searched using standard protein translation. Then, unidentified spectra were assigned to identified spectra based on mass differences by potential mistranslations to reduce the search space. Subsequently, for each combination of identified peptide and unidentified spectrum, a quasi-minimalistic database search was performed using the unidentified spectrum and a small peptide database generated from the peptide of the corresponding identified spectrum mutated as far as the mass shift allowed. This way, the problem of searching against an impractically big database was reduced to thousands of searches against minimum databases.

The database search algorithm has no a priori knowledge about the “correct” translation and therefore treats all spectrum matches equivalent during scoring and filtering. In addition to applying a of 1% for global quality filtering, we filtered spectrum matches of peptides containing CUG-translated amino acids by requiring residues at CUG positions to be supported by b- and/or y-type fragment ions on both sides. The majority of these amino acids, 89 to 95% depending on sample, are part of extended chains of b-/y-type supported amino acids (Additional file 1).

### Unambiguous translation of CUG as serine in all CTG-clade yeasts

**Fig. 2:**
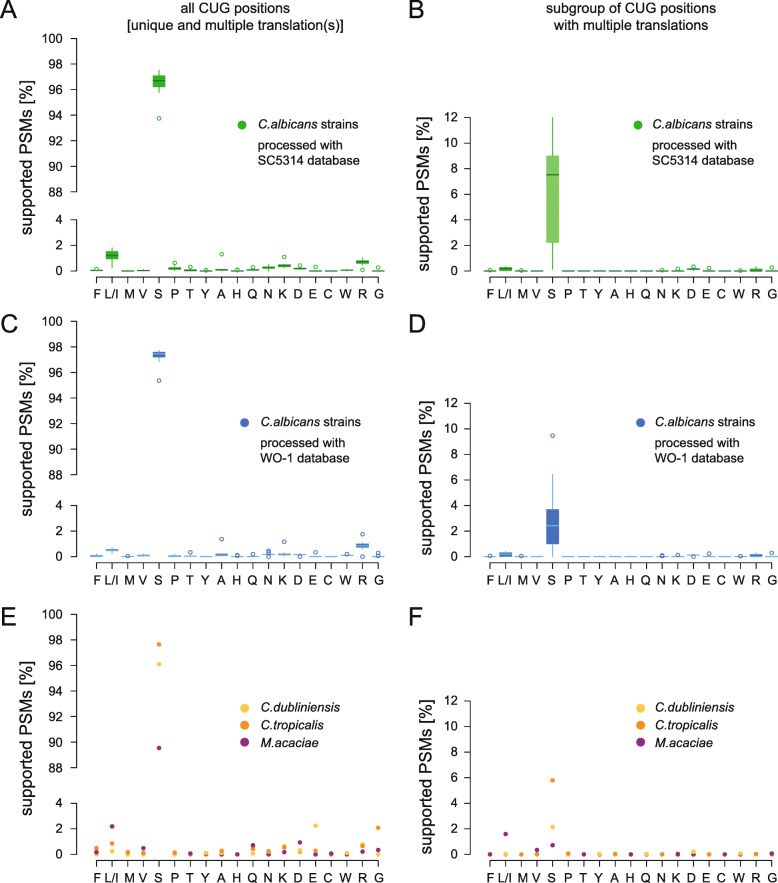
Percentage of PSMs containing a b-/y-type fragment ion supported CUG codon by CUG translation. **A** The MS/MS data of the nine *C. albicans* samples were processed with a database derived from the allele-resolved SC5314 genome annotation. All PSMs containing CUG positions supported by b-/y-type fragment ions (= supported PSMs; Table 1) were collected and their distribution plotted with respect to the amino acid found at the CUG position. **B** The plot shows the distribution of the subset of supported PSMs covering only those CUG positions, where PSMs with at least two different amino acids at the CUG positions were found. **C, D** The MS/MS data of the nine *C. albicans* samples were processed with a database derived from the WO-1 genome annotation. PSM selection and plotting as in **A** and **B**. **E, F** Data from the *C. dubliniensis*, *C. tropicalis*, and *M. acaciae* samples for comparison. The prevalent CUG translation, serine, is found in almost all of the corresponding PSMs. The numbers of CUG translations other than serine are higher for the allele-resolved *C. albicans* SC5314 database analyses (**A**) and (**B**) than for the pseudo-haploid WO-1 database analyses (**C**) and (**D**) indicating strain differences and haplotype merging effects. (Mis-)translation into leucine/isoleucine is, contrary to what is expected based on a suspected 3% mischarging of ${\mathrm{tRNA}}_{\mathrm{CAG}}^{\mathrm{Ser}}$ with leucine, no more prevalent than mistranslation into any other amino acid (**B**) and (**D**)

Analysing 11,084,524 MS/MS spectra using this approach, we recovered 10 to 18% (5 to 12% with b-/y-type support) of the total CUG positions in the genomes, spread across 1000 to 2400 genes (Table 1, Additional file 2). These values are similar to those obtained from the previously analysed yeasts *B. inositovora* and *C. lusitaniae*. CUG position coverage correlated with the number of measured and processed spectra, which is expected given very similar numbers of CUG codons in the genomes of *C. albicans*, *C. dubliniensis*, and *C. tropicalis* as compared to 1.5- and 2-fold more in the genomes of *B. inositovora* and *C. lusitaniae*, respectively. When comparing observed CUG codon translations we found 99% ± 1% (on average 485 positions, numbers from here on refer to b-/y-type supported positions) CUG codon positions in *C. albicans* to be translated unambiguously, namely 95% (average of 464 positions) translated into serine and 4% (average of 21 positions) into any other amino acid (including, but not exclusively, leucine or isoleucine; Fig. 2A). The CUG codons translated as leucine or isoleucine are most affected by processing the data against diploid (SC5314 strain) versus pseudo-haploid (WO-1 strain) annotations indicating strain and/or allele differences and not mistranslations (more details below; Fig. 2C). At the remaining 1% (average 4 positions) CUG positions, peptides with at least two different CUG codon translations were found. The unambiguous serine-translated CUG codon positions were supported by an average of 1961 PSMs per sample (on average 4 PSMs per position; PSM = peptide spectrum match). At ambiguous positions, 12 times more PSMs with CUG translated as serine compared to all other amino acids were found (Fig. 2B). Notably, only an average of 4 (0.2%) PSMs were found with CUG codons translated as leucine or isoleucine at ambiguous positions (average one position, or 0.2%).

**Table 1:** Number of obtained PSMs and proteins and CUG positions covered. If not indicated otherwise, numbers include positions both with and without direct b-/y-type support

Yeast	Strain	Growth form	Protein database	# PSM with CUG	# PSMs with supported CUG	# CUG positions covered	# supported CUG positions covered	% supported positions	# identified proteins with CUG	% identified proteins with CUG	CUG position recovery [%]
*Candida albicans*	DSM70014	Yeast	SC5314	3510	2206	345	243	70.43	1406	17.46	9.85
*Candida albicans*	SC5314	Yeast	SC5314	1761	1024	658	413	62.77	2151	26.71	10.69
*Candida albicans*	SC5314	Hyphal	SC5314	6368	3549	1148	719	62.63	2687	33.37	14.1
*Candida albicans*	EU0006	Yeast	SC5314	3801	2078	762	469	61.55	2306	28.64	11.3
*Candida albicans*	EU0006	Hyphal	SC5314	4021	2365	934	608	65.1	2503	31.09	12.38
*Candida albicans*	EU0009	Yeast	SC5314	4838	2754	931	599	64.34	2477	30.76	12.59
*Candida albicans*	EU0009	Hyphal	SC5314	4405	2467	925	610	65.95	2515	31.23	12.04
*Candida albicans*	EU0075	Yeast	SC5314	3284	1611	756	413	54.63	2240	27.82	11.47
*Candida albicans*	EU0075	Hyphal	SC5314	3501	1762	876	499	56.96	2446	30.38	11.7
*Candida albicans*	DSM70014	Yeast	WO-1	3195	2048	329	223	67.78	1197	30.27	11.63
*Candida albicans*	SC5314	Yeast	WO-1	1501	855	592	371	62.67	1756	44.41	12.47
*Candida albicans*	SC5314	Hyphal	WO-1	5414	3015	1003	644	64.21	2136	54.02	16.25
*Candida albicans*	EU0006	Yeast	WO-1	3436	1862	719	435	60.5	1861	47.07	13.89
*Candida albicans*	EU0006	Hyphal	WO-1	3619	2093	888	572	64.41	1999	50.56	15.59
*Candida albicans*	EU0009	Yeast	WO-1	4412	2423	882	557	63.15	1981	50.1	15.67
*Candida albicans*	EU0009	Hyphal	WO-1	4066	2217	872	570	65.37	2005	50.71	15.06
*Candida albicans*	EU0075	Yeast	WO-1	2957	1424	709	381	53.74	1795	45.4	14.24
*Candida albicans*	EU0075	Hyphal	WO-1	3166	1568	834	471	56.47	1959	49.54	14.79
*Candida dubliniensis*	CBS 7987	Yeast		966	466	232	121	52.16	1003	25.61	10.02
*Candida tropicalis*	DSM 24507	Yeast		3032	1827	378	262	69.31	1247	31.45	12.64
*Millerozyma acaciae*	CBS 5656	Yeast		4962	2895	1631	1111	68.12	2418	67.62	17.85
*Babjeviella inositovora*	NRRL Y-12698	Yeast		4937	2348	1211	930	76.8	2122	47.47	17.34
*Clavispora lusitaniae*	ATCC 42720	Yeast		5803	2715	2360	1835	77.75	2843	56.95	15.87

In *C. dubliniensis*, 94% of the PSMs mapped to unambiguous positions and only a single CUG position with ambiguous translation was found (Fig. 2E, F). Twenty-seven PSMs contained this position translated as serine and one PSM contained the same position as aspartate. Similarly, in *M. acaciae* no CUG codon position ambiguously translated with leucine or isoleucine was found. In *C. tropicalis*, 1787 PSMs mapped peptides with CUG codons translated as serine, and 40 (2.2 %) PSMs contained leucine or isoleucine at the CUG codon position. Eleven from the latter PSMs mapped to codons where no other translation than leucine or isoleucine was found. The other 29 PSMs mapped to a single CUG codon position in the ubiquilin gene. At the same codon position, a minority of nine PSMs had the CUG translated as serine. A phased diploid genome assembly of *C. tropicalis* is not available, which could reveal whether the dual translation is the result of different codons in the two alleles. Although ${\mathrm{tRNA}}_{\mathrm{CAG}}^{\mathrm{Ser}}$ of most of the analysed yeasts differ from the *C. albicans* ${\mathrm{tRNA}}_{\mathrm{CAG}}^{\mathrm{Ser}}$, these in vivo data show that individual sequence differences do not correlate with and result in a supposed CUG mistranslation into leucine/isoleucine. *C. dubliniensis* with an identical ${\mathrm{tRNA}}_{\mathrm{CAG}}^{\mathrm{Ser}}$ compared to *C. albicans* did not show any ambiguity towards leucine.

### Amino acids other than serine at CUG positions—allele differences

**Fig. 3:**
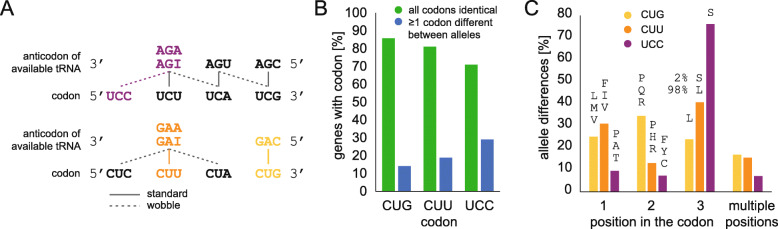
Differences at CUG, CUU and UCC codons between alleles. **A** Schematic view of serine and leucine codon boxes and available tRNAs. **B** Genes of the *C. albicans* SC5314 genome annotation with haplotype assignment (tagged A/B) and identical length were compared with respect to CUG, CUU, and UCC codons. Each of the three codons was analysed separately. For simplicity, alleles were only distinguished into those with identical codons at all occurrences of the respective codon, and those where the alleles differ at least one of the codons (if there are multiple in the genes). **C** Analysis of the differences at all codon sites where the alleles differ. The differences at codons are split for the three codon positions and the rest combining multiple differences. The corresponding amino acids at the other allele are given in smaller letters for orientation. The ratio for the differences identified at the third position of the CUU codon (CUA/CUC versus CUG) is given in the plot, and the exact distribution of the other codon sites are available in Additional file 3 Table S3

Across the nine *C. albicans* samples (five strains, four of these each in yeast and hyphal growth forms), 423 CUG codon positions were found with the CUG codons not translated by serine (Fig. 2A). These translations can have four reasons: (1) the strains analysed could have mutations at the respective positions compared to the gene annotations (search databases were generated from the annotations of the SC5314 and WO-1 strains); (2) the differences could result from alleles, which might not be present in the gene annotations (the WO-1 is a pseudo-haploid genome assembly); (3) the differences might be the result of codon mistranslation while the correctly translated peptide is missing; (4) there might be differences due to sequencing errors or low genome coverage in the reference strains. Peptides with other translations than serine due to sequencing errors, strain differences, and allelic expression should be present in multiple samples, while peptides resulting from random ribosomal mistranslation should be unique to a single sample. The presence of a phased diploid *C. albicans* genome assembly and annotation [21, 22] allows linking the genome and the proteome. In total, 3632 of the 5804 genes with same length differ between the alleles (Additional file 3 Table S2). For comparison, we also analysed the related codons CUU and UCC (Fig. 3). The UCC codon is decoded by the non-cognate ${\mathrm{tRNA}}_{\mathrm{AGA}}^{\mathrm{Ser}}$, the CUU codon by the cognate ${\mathrm{tRNA}}_{\mathrm{AAG}}^{\mathrm{Leu}}$ (Fig. 3A). A total of 1303 genes with CUG codons (1846 with CUU and 1851 with UCC) also have CUG (CUU/UCC) codons at the same position in the allele. In total, 215 genes (430 with CUU and 759 with UCC) differ at 244 CUG codons (576 CUU and 1000 UCC codons) between alleles (Fig. 3B). This means that genes with UCC are more likely to differ at the UCC codon with their allele and that UCC codons are more likely to differ in general, compared to CUG and CUU. The differences at the UCC codons correspond well with the general observation that codons evolve faster at the third codon position (Fig. 3C). This is in strong contrast to the CUG and CUU codons. Mutation at the third position of CUU can also result in CUG, which is, however, translated into the physicochemically different serine. This mutation might disturb protein folding and would be selected against. Correspondingly, almost all differences at the third codon position result in CUC and CUA (Fig. 3C, Additional file 3 Table S3). The disturbance of the third codon position might be the reason for the increased number of differences at the first codon position, compared to UCC. For the CUG codon, the third codon position is the least favoured with regard to allelic differences. Most differences between the alleles are at the second codon position leading to proline, hydrophilic glutamine, and charged arginine, which are likely more tolerable at the protein surface where most of the serines are located. In the proteogenomics data, we did not find any peptide covering a CUU codon where the CUU position is part of an allelic difference (Additional file 3 Table S4). For the CUG codon, we found three CUG positions where peptides matching the other allele were found. Two positions match CAG codons (for glutamine) and one position matches a UUG codon (for leucine). The CAG codons were found in single samples, the UUG codon was found in six of the nine samples. None of the three positions was covered by peptides with a serine at the CUG position. Thus, the majority of the peptides with leucine at CUG positions (see Fig. 1A) are not the result of translational ambiguity but the result of the allele-specific translation of the allele with the UUG codon. For the UCC codon, we found peptides matching a single allele with an AGC codon and about 40 alleles with UCU, UCA, or UCG codons at the UCC codon position (Additional file 3 Table S4). All of this indicates strong allele-specific translation as has been found elsewhere [23].

### Amino acids other than serine at CUG positions

**Fig. 4:**
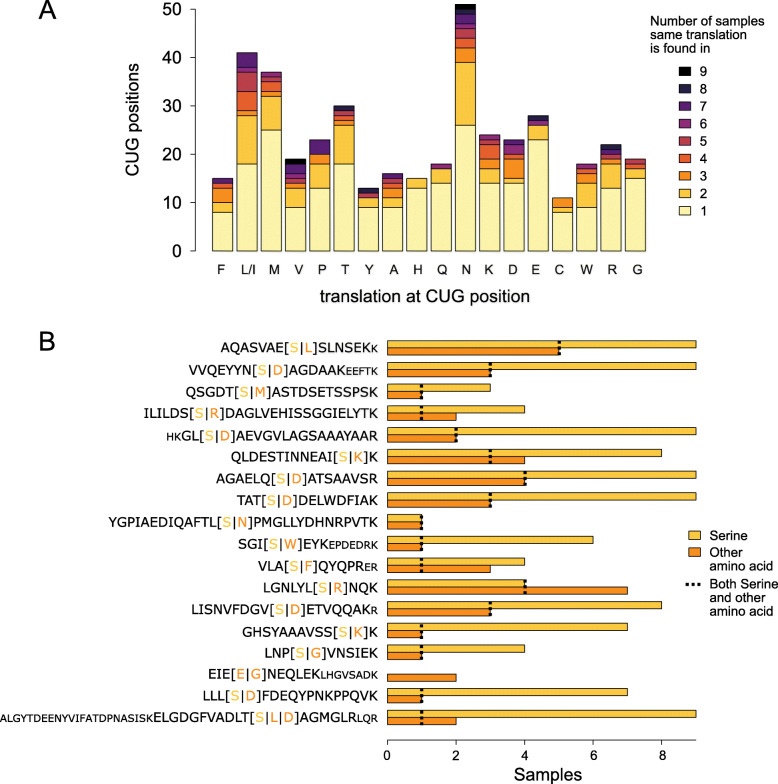
CUG codon positions with translations other than serine. **A** The number of CUG codon positions at which only amino acids other than serine were found separated by their identification in only one or multiple of the nine samples (five *C. albicans* strains, yeast and hyphal growth forms for four strains). **B** The plot lists the eighteen CUG codon positions for which peptides with different CUG translations were found. Translations are distinguished into “serine” and “any other amino acid”. Alternative translations are indicated in brackets in the peptide sequences. The number of samples in which multiple translations were found in a single sample is indicated with a dotted line. For peptides [1, 3] (read from the bottom), different non-serine translations were found in different samples. Peptides with missed cleavages are indicated by lowercase letters

At 165 (39.0%) CUG codon positions, the identical translation was found in at least two different samples indicating strain differences (Fig. 4A, Additional file 4). At the other 258 CUG positions, peptides found were unique to one of the nine samples. However, only 7.0% of these unique peptides had leucine (or isoleucine) at the CUG position implying that if these were the result of mistranslation (e.g. from leucine-mischarged ${\mathrm{tRNA}}_{\mathrm{CAG}}^{\mathrm{Ser}}$), mistranslation by other amino acids must be more prevalent.

Combining all nine samples there are 18 CUG positions at which at least two peptides with the CUG translated into different amino acids were found (Fig. 4B). A total of 2419 PSMs support the serine translation, and 127 PSMs the respective other translations. Because of experimental sampling depth, only six of these CUG positions are covered in every of the nine samples, and in every single sample only two to nine of these 18 CUG positions are covered by multiple peptides with different translations. At ten of these 18 positions, the combination of serine and another amino acid (leucine or isoleucine in one case) was found in at least two samples indicating allelic expression. At six positions, ambiguous translation with serine and another amino acid (but not leucine or isoleucine) was found in only a single sample. At one position, ambiguous translation by glutamate and glycine but not serine was found, and at another position, three amino acids, serine, aspartate and leucine or isoleucine, were found. In summary, serine/leucine (or isoleucine) ambiguity was found at only a single CUG position, which rather implies low-level mistranslation than CUG ambiguity.

On average, 2073 (0.86%) of the PSMs of each *C. albicans* sample span a CUG codon. If a 3% mischarging of the ${\mathrm{tRNA}}_{\mathrm{CAG}}^{\mathrm{Ser}}$ by leucine was assumed as proposed by earlier studies [8, 9], an average of 62 PSMs with the CUG codon translated by leucine are to be expected per sample. This is in sharp contrast to the experimentally found average of only 17 PSMs that further separate into those unique for the position (no serine translation at the position found, most likely allele-specific expression as described above) and those at the two positions where serine and leucine or isoleucine were found at the same time.

### Using controlled bacterial contamination as reference for the detection level

**Fig. 5:**
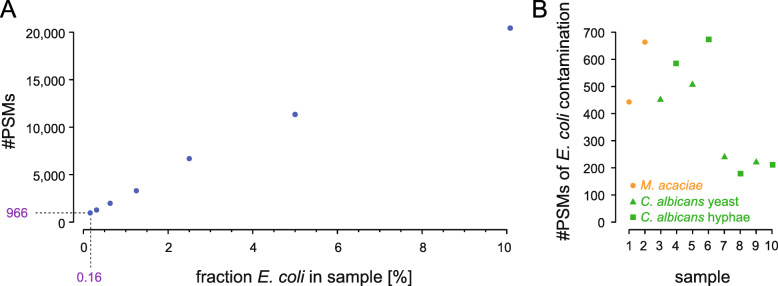
Analysis of *E. coli* contamination level. **A** Number of PSMs matching *E. coli* proteins in an otherwise *M. acaciae* sample by fraction of *E. coli* in the sample. **B** Number of *E. coli* PSMs found in samples that were not purposely mixed with *E. coli*. Samples 1 and 2 are *M. acaciae* samples used as control to determine the *E. coli* contamination level in a supposed “*E. coli* free” sample. Samples 3 to 8 are *C. albicans* strains SC5314, EU0006, EU0009, and EU0075, each in yeast and hyphal growth form. The number PSMs found for background *E. coli* contamination is below the mixture sample with 0.16% *E. coli* and 99.84% *M. acaciae*. The number of *E. coli* PSMs was normalized to 100,000 PSMs for each sample

Within a proteomics approach, it is difficult to determine an absolute detection level. Because of the chromatographic methods, some peptides might be disfavoured compared to others and appear at different levels although injected with same amount. Precursor mass scanning favours the identification of peptides with so far undetected masses, which causes the more abundant peptides to appear less abundant than they are. Here, we thought about a method to determine the relative level at which we could detect peptides. Rather than adding synthetic peptides with their inherent problem of response factors, we went for adding the lysate of bacteria. This way we simulate a mix of peptides comparable to the natural mix of peptides covering a certain codon, here CUG. For easier handling, we mixed *M. acaciae* with *Escherichia coli*. As a starting point, a mixture of 90% (by protein concentration) *M. acaciae* cell lysate and 10% (by protein concentration) *E. coli* cell lysate was prepared. This sample was diluted by half with *M. acaciae* cell lysate in several steps to a final concentration of 0.16% *E. coli* (Fig. 5A). At this low concentration, *E. coli* proteins could well be detected covered by almost 1000 PSMs. For comparison, we analysed the supposedly “*E. coli* free” *M. acaciae* sample and several *C. albicans* samples combining the yeast-specific databases with the *E. coli* database. This analysis revealed a considerable fraction of *E. coli* contamination in all samples although highest standards were followed for clean work (Fig. 5B). The fraction of PSMs covering CUG codons is between 1.3 and 1.7% for *C. albicans* and between 2.0 and 3.2% for *M. acacia*. These fractions together with respective numbers of PSMs fit to the *E. coli* contamination data (compare Table 1 and Fig. 5A). Extrapolating the *E. coli* data, a hypothesized 5% misincorporation at CUG codons with leucine would correspond to 0.06 to 0.09% *E. coli* contamination and should, therefore, result in about 600 PSMs with CUG translated as leucine. It should have been well possible to observe even half of these PSMs in our data.

### Are other codons as unambiguous as the CUG codon?

**Fig. 6:**
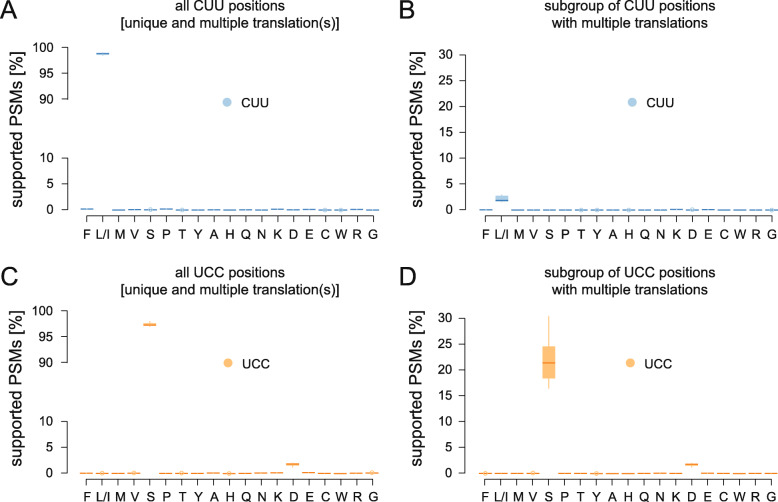
Percentage of PSMs containing a b-/y-type fragment ion supported CUU (leucine) or UCC (serine) codon by translation. **A** The MS/MS data of the nine *C. albicans* samples were processed with a database derived from the WO-1 genome annotation. All PSMs containing CUU positions supported by b-/y-type fragment ions (= supported PSMs; Table 1) were collected and their distribution plotted with respect to the amino acid found at the CUU position. **B** The plot shows the distribution of the subset of supported PSMs covering only those CUU positions, where PSMs with at least two different amino acids at the CUU positions were found. **C, D** PSM selection and plotting as in **A** and **B**, but based on the UCC codon

With a codon usage of 0.42%, CUG belongs to the rarely used codons in the *C. albicans* genome annotation [22]. To determine whether the observed unambiguous CUG codon translation depends on the global codon frequency and/or the type of amino acid, we analysed the CUU leucine and the UCC serine codons, which are used with frequencies of 0.28% and 0.87% in the genome, respectively. Although their global genomic codon usage differs by a factor of three as does the number of PSMs covering the respective codons, the number of CUU and UCC codons covered with PSMs was almost identical (WO-1 based database; Additional file 5). Strikingly, about four to five times more CUU and UCC codons than CUG codons were found to be covered by PSMs with the respective position(s) being supported by b-/y-type fragment ions. This indicates that CUU and UCC codons, although rare globally, are present in commonly expressed genes while the CUG codons are rather enriched in genes with low or no detectable expression level. Similar to CUG, both CUU and UCC codons are predominantly covered by PSMs with their respective standard translation, namely 98.78% of supported PSMs covering CUU translated as leucine (Fig. 6A) and 97.38% of supported PSMs covering UCC translated as serine (Fig. 6C; Additional file 6). 0.73% and 5.09% of the CUU and UCC codon positions, respectively, are covered by peptides with at least two different translations. On average, 2.16% and 22.01% of PSMs covering the CUU and UCC codons translated as leucine and serine, respectively, were found at positions where PSMs with other translations of the codon were also observed showing dominance of the standard translation (Fig. 6B, D). Together, this indicates that the amino acid positions encoded by UCC serine codons tolerate about six times higher levels of mistranslation than those encoded by the CUU leucine codon, likely because serine amino acid positions are enriched at the surface of proteins and are generally less conserved than leucine amino acid positions [4, 24]. This analysis of other, related codons demonstrates that the CUG codon is translated in *C. albicans* as unambiguously as other codons. If CUG was supposed to be translated ambiguously, a similar ambiguity would have to be assumed for other codons as well.

### Unambiguous translation of CUG as serine in *C. albicans* yeast and hyphal growth forms

To investigate whether the reported CUG ambiguity is strain or growth form dependent, we tested five strains, DSM70014, SC5314 (clade 1), and three genetically distinct clinical isolates EU0006 (clade 2), EU0009 (clade 12), and EU0075 (clade 4) [25]. The latter four strains were grown in yeast and hyphal growth form. All analyses resulted in the same unambiguity of the CUG translation (Table 1, Fig. 1).

## Discussion

Our unbiased, statistical evaluation of proteome data suggests that CTG-clade yeasts do not translate the CUG codon ambiguously in vivo. Although there is a measurable level of CUG mistranslation, this level is (i) similar to that of the leucine CUC and serine UCC codons, (ii) similar in six CTG-clade species covering different substitutions of the conserved guanosine nucleotides adjacent to the CAG anticodon in ${\mathrm{tRNA}}_{\mathrm{CAG}}^{\mathrm{Ser}}$, and (iii) similar in yeast and hyphal growth forms of *C. albicans*. Last but not least, mistranslation of CUG into leucine (or isoleucine) is not preferred, although this would be expected if the ${\mathrm{tRNA}}_{\mathrm{CAG}}^{\mathrm{Ser}}$ were partially mischarged by leucine. Instead, several other amino acids were found at similar levels at CUG codon positions.

How do our findings relate to previous reports of slight CUG mistranslation into leucine? In 1997, tRNA pools were purified from *C. cylindracea* and *Candida zeylanoides* and radioactively labelled immediately [8]. The ${\mathrm{tRNA}}_{\mathrm{CAG}}^{\mathrm{Ser}}$ were subsequently pulled out of the mixture using a solid-phase attached DNA probe. This step is crucial because the DNA probe could be selective enough to extract the *C. cylindracea* ${\mathrm{tRNA}}_{\mathrm{CAG}}^{\mathrm{Ser}}$ but not to quantitatively exclude all *C. zeylanoides* Leu-tRNA. The entire approach allows for accumulating contaminations at multiple steps and control experiments using other tRNAs are missing. This is a very important note because the authors proposed that their DNA probe was selective and excluded other tRNAs, while, as a result of their study, the endogenous leucyl-tRNA synthetase was suggested to not be completely selective against the ${\mathrm{tRNA}}_{\mathrm{CAG}}^{\mathrm{Ser}}$. In the same study, the authors performed a genetic rescue experiment introducing a plasmid encoding the *Saccharomyces cerevisiae URA3* gene, which contains a leucine essential for activity, into *Candida maltosa* [8]. While *C. maltosa* was not viable with a serine codon at the essential leucine position, weak growth was observed in case of a CUG codon. However, experiments such as this performed under strong selection do not allow distinguishing translation by mischarged ${\mathrm{tRNA}}_{\mathrm{CAG}}^{\mathrm{Ser}}$ from mistranslation by non-cognate Leu-tRNAs. The latter argument might also explain the observed incorporation of leucine into a reporter peptide in vivo [9]. In that study, a peptide containing a serine encoded by a CUG codon was fused to a reporter protein, the protein overexpressed in *C. albicans*, then purified and in-gel digested, and the resulting peptides identified and quantified using high-pressure liquid chromatography and tandem mass spectrometry. As control, the authors analysed potential mistranslation of lysine AAA and aspartate GAU codons by near-cognate Asn- and Glu-tRNAs, respectively [9]. However, yeast genomes only contain the near-cognate ${\mathrm{tRNA}}_{\mathrm{GUU}}^{\mathrm{Asn}}$ and ${\mathrm{tRNA}}_{\mathrm{YUC}}^{\mathrm{Glu}}$, which do not allow translation of AAA and GAU codons by standard or wobble base pairing rules [7, 26]. Therefore, such mistranslations were, in fact, not observed. In contrast, the serine CUG codon can be mistranslated by the non-cognate ${\mathrm{tRNA}}_{\mathrm{CAA}}^{\mathrm{Leu}}$ establishing a C•A mispair at the first codon—third anticodon position [27]. It is also known that stress increases mistranslation levels in general [28, 29] and likely this is what the authors observed when co-expressing a *S. cerevisiae* mutant ${\mathrm{tRNA}}_{\mathrm{CAG}}^{\mathrm{Leu}}$ [9]. Unfortunately, control experiments using the identical peptide but testing other codons for potential mistranslations caused by wobble mis-pairings were not performed there. Also, mistranslation by leucine versus mistranslation by isoleucine cannot be distinguished.

It could be possible that we did not observe CUG codons mistranslated as leucine because they were not in the prepared cell lysates. CUG codons in *C. albicans* and also the other CTG-clade yeasts are, to considerable extent, found at conserved serine positions in proteins [4, 24] but never at conserved leucine positions. Thus, it could be possible that proteins with leucines randomly introduced at important serine positions were immediately degraded. However, *C. albicans* with more than 25% of the CUG codons artificially translated as leucines was shown to be viable [9, 15], which suggests that misincorporation at the level of ribosomal mistranslation or minor global percentages should well be tolerated. Otherwise, this would contradict the hypothesis of a global 3% leucine translation of CUG given the experimental setup of the studies reporting this rate. If degradation of proteins with leucines at CUG positions happened in our study, the same should have happened in the other studies as well and ambiguity should have never been reported. Alternatively, CUGs translated to leucine could be enriched in cell wall proteins that were not present in the cell lysate. However, we are not aware of any mechanism that would preferentially select Ser-tRNAs charged with leucine (as suggested by the ${\mathrm{tRNA}}_{\mathrm{CAG}}^{\mathrm{Ser}}$ mischarging hypothesis) for synthesizing cell wall and secretome proteins while excluding these mischarged ${\mathrm{tRNA}}_{\mathrm{CAG}}^{\mathrm{Ser}}$ for soluble proteins. Similar to the degradation argument above, CUG translation ambiguity should have not been observed in the other studies that also only analysed soluble proteins.

Serine and leucine incorporation at CUG positions could be context dependent. However, we are not aware of any mechanism by which ribosomes could select mischarged ${\mathrm{tRNA}}_{\mathrm{CAG}}^{\mathrm{Ser}}$ instead of correctly charged ${\mathrm{tRNA}}_{\mathrm{CAG}}^{\mathrm{Ser}}$ in a sequence-dependent context similar to that of the selenocysteine incorporation at stop codons. In the latter case, a tRNA is selected instead of a release factor, which are completely different entities compared to correctly charged and mischarged ${\mathrm{tRNA}}_{\mathrm{CAG}}^{\mathrm{Ser}}$, which are identical except for a hydroxyl and an isopropyl group at the amino acid side chain.

Are our proteogenomics experiments sufficiently reliable to determine codon mistranslation? There are several findings that support our conclusions. First, the observed CUG codon ambiguity is below the level at which we detected bacterial contamination in the supposedly bacteria-free samples. We have taken every effort to prepare pure samples and high experimental standards are indeed a requirement for working with *C. albicans*. Nevertheless, *E. coli* proteins could be detected at very low levels. This indicates that the approach allows detecting peptides at a very low level, which is, in fact, in the range of expected ribosomal mistranslation. Second, we did find some CUG codon positions with translations other than serine, and even positions with multiple different translations. This ambiguity is either caused by mistranslation or by sequence differences between the alleles. If we assumed no allelic differences or expression of only one of the two alleles in this diploid species, the maximum possible mistranslation level would then correspond to the observed level of other amino acids found at CUG codon positions. However, explaining the non-serine translation of CUG codons by strain and allelic differences is much more likely because most of these translations were found in multiple samples and because non-serine translations decreased considerably when analysing the data with the pseudo-haploid WO-1 annotation instead of the diploid SC5314 annotation. Third, we found similar levels of ambiguity for other rare leucine and serine codons which indicates that the observed mistranslation level corresponds to the general ribosomal mistranslation rate. This is further supported by observation of similar mistranslation rates in other yeasts [3, 4]. If the assumed 3–5% mistranslation of CUG codons by leucine were below the detection level of our approach, the background ambiguity by ribosomal mistranslation would be at an even higher level.

## Conclusions

Our findings suggest that *C. albicans* does not decode CUG ambiguously. It has been suggested that CUG decoding ambiguity causes phenotypic diversity and, therefore, allows *C. albicans* to explore available ecological landscapes [15]. In this study, *C. albicans* cells were exposed to stress by expressing a recombinant ${\mathrm{tRNA}}_{\mathrm{CAG}}^{\mathrm{Leu}}$. However, exerting stress in general might lead to misacylation of many tRNAs, induce a general higher ribosomal mistranslation level, or disturb the allele-specific transcription and translation thus causing phenotypic diversity to adapt to changing environments. Our data suggest that the proposed misacylation of the ${\mathrm{tRNA}}_{\mathrm{CAG}}^{\mathrm{Ser}}$ by leucine might be as prevalent as every other misacylation and, if at all, only be one of many reasons causing phenotypic diversity.

## Methods

### Growth and lysis of fungal cells

*Candida dubliniensis* CBS 7987 (CD36) and *Millerozyma acaciae* CBS 5656 (JCM 10732) were obtained from the CBS-KNAW culture collection of the Westerdijk Fungal Biodiversity Institute, Netherlands. The other strains are part of the yeast collection of the Institute of Microbiology of the University Medical Center Göttingen. *C. albicans* DSM70014, *C. dubliniensis* CBS 7987, and *Candida tropicalis* DSM 24507 were grown in YEPD medium (containing [% w/v]: bacto peptone 2.0; yeast extract 1.0; glucose 2.0) at 37 °C. *M. acaciae* was grown in YEPD medium at 30 °C. Cells were harvested and lysed exactly as described earlier [4]. Proteins were resuspended in SDS sample buffer and resolved on 4–12 % gradient SDS-PAGE.

MLST-typed [25] *C. albicans* isolates SC5314 (dST 52, clade 1), EU0006 (dST 1321, clade 2), EU0009 (dST299, clade 12), and EU0075 (dST124, clade 4) were subcultured three times on SAB agar after thawing. For production of yeast form cells, 50 ml Lee’s medium [30] with a pH of 4.5 were inoculated with a fresh colony and incubated at 30 °C and 140 rpm in an orbital shaker overnight. This overnight culture was then diluted to an OD of 1.0 in 150 ml Lee’s pH 4.5 and grown at 30 °C and 170 rpm in an orbital shaker. Hyphae form cells were produced starting from the above yeast overnight culture. Cells were treated identically as above except for using Lee’s medium with pH 6.5 and shaking at 37 °C. Cells from 100 ml fungal culture were harvested during exponential phase (after ~ 6 h) by centrifugation at 4700×*g* for 5 min. The pellets were washed in 10 ml fresh Lee’s medium (pH 4.5 or 6.5, respectively). Subsequently, 1 ml aliquots were taken from the cell suspension, centrifuged at 15,600×*g*, the supernatants were discarded and cells were shock frozen in liquid nitrogen and stored at − 20 °C. For SDS-PAGE, cell pellets were defrosted, 50 μl of loading buffer added, and samples vortexed for 10 s. For mechanical cell lysis, glass beads were added and samples were passed twice for 20 s in a FastPrep machine (speed setting 4.0). In between, samples were cooled on ice for 1 min. Proteins were resolved on 12% SDS-PAGE.

### Genome assemblies and annotation

Genome annotations for *C. albicans* SC5314 A22 (assembly 22), *C. albicans* WO-1, *C. dubliniensis* CD36, and *C. tropicalis* MYA-3404 were obtained on 18/11/2018 from the Candida Genome Database [22]. The *M. acaciae* JCM 10732 genome assembly was downloaded from GenBank with the accession BCKO01000000 [31]. *M. acaciae* JCM 10732 genes were predicted with AUGUSTUS [32] using the parameter “genemodel = complete”, the gene feature set of *Candida albicans*, and the standard codon translation table.

### Dilution of *M. acaciae* samples with *E. coli*

Protein concentration of *M. acaciae* and *E. coli* cell lysates was determined using the Biuret method. As starting sample, a mixture of 90% *M. acaciae* and 10% *E. coli* was produced, subsequently an aliquot of this sample mixed with an identical volume of the *M. acaciae* cell lysate, and the latter process of diluting the *E. coli* concentration by half repeated several times. This way, pipetting errors were reduced as much as possible.

### Mass spectrometric sequencing

SDS-PAGE-separated protein samples were processed as described by Shevchenko et al. [33]. The resuspended peptides in sample loading buffer (2% acetonitrile and 0.05% trifluoroacetic acid) were separated and analysed on an UltiMate 3000 RSLCnano HPLC system (Thermo Fisher Scientific) coupled online to either a Q Exactive HF or an Orbitrap Fusion mass spectrometer (Thermo Fisher Scientific). Firstly, the peptides were desalted on a reverse phase C18 pre-column (Dionex 5 mm long, 0.3 mm inner diameter) for 3 min. After 3 min, the pre-column was switched online with the analytical column (30 cm long, 75 μm inner diameter) prepared in-house using ReproSil-Pur C18 AQ 1.9 μm reversed phase resin (Dr. Maisch GmbH). The peptides were separated with a linear gradient of 5–45% buffer (80% acetonitrile and 0.1% formic acid) at a flow rate of 300 nl/min (with back pressure 500 bars) over 58 min gradient time. The pre- and main column temperatures were maintained at 50 °C. In the Q Exactive Plus, the MS data were acquired by scanning the precursors in mass range from 350 to 1600 m/z at a resolution of 70,000 at m/z 200. Top 20 precursor ions were chosen for MS2 by using data-dependent acquisition (DDA) mode at a resolution of 17,500 at m/z 200 with maximum IT of 50 ms. In the Q Exactive HF, the MS data were acquired by scanning the precursors in mass range from 350 to 1600 m/z at a resolution of 60,000 at m/z 200. Top 30 precursor ions were chosen for MS2 by DDA mode at a resolution of 15,000 at m/z 200 with maximum IT of 50 ms. Data were measured on Q Exactive HF instrument except for *M. acaciae* experiments which were measured on Orbitrap Fusion.

### Mass spectrometry data analysis

Data analysis and search were performed using MaxQuant v.1.6.0.1 (samples Calbicans_WO1_1, Calbicans_SC5314_1, *C. dubliniensis*, *C. glabrata*, and *C. tropicalis*) and MaxQuant v.1.6.5.0 (all other samples) as search engine with a global and a peptide-level 1% FDR. To obtain peptide mappings free of codon translation bias, 19 replicates for each genome annotation were generated with the codon of interest (CUG, CUU, UCC) translated as different amino acid in each replicate (translation as isoleucine being omitted as leucine and isoleucine are indistinguishable through MS/MS). To reduce database size and redundancy, predicted proteins were split at lysine and arginine residues into peptides resembling trypsin proteolysis. Peptides containing the respective codons were fused with up to two peptides in N- and C-terminal direction (depending on the codon being at the terminal ends of the protein) so that codon-containing fragments can be detected with up to two missed cleavages. The remaining peptides were fused back together as long as they formed consecutive blocks. Duplicate peptides (originating from peptide-blocks without the codon of interest) were removed. Search parameters for searching the precursor and fragment ion masses against the databases were as described in Mühlhausen et al. [4]. To claim codon translations with high confidence, we determined whether the respective codon positions are supported by b- and y-type fragment ions at both sides allowing determination of the amino acids’ mass. Only those positions were regarded as fully supported by the data.

## Supplementary Information

**Additional file 1 **: **Table S1**. MS/MS data processing statistics.

**Additional file 2.** Raw and additional data relevant to Figure 2.

**Additional file 3 **: **Tables S2-S4**. **Table S2**: Gene and codon numbers in the annotation of the diploid *Candida albicans* strain SC5314. **Table S3**: CTG, CTT and TCC codons and the corresponding codons in the other allele in the annotation of the diploid *Candida albicans* strain SC5314. **Table S4**: Identification of peptides with codons corresponding to CTG or TCC in the other allele in the annotation of the diploid *Candida albicans* strain SC5314.

**Additional file 4.** Raw and additional data relevant to Figure 4.

**Additional file 5 **: **Tables S5-S7**. **Table S5**: MS/MS data processing statistics against CTT and TCC databases. **Table S6**: Number of obtained PSMs and proteins and CTT positions covered. **Table S7**: Number of obtained PSMs and proteins and TCC positions covered.

**Additional file 6.** Raw and additional data relevant to Figure 6.

## Data Availability

The mass spectrometry data from this study have been submitted to the ProteomeXchange Consortium (http://proteomecentral.proteomexchange.org) via the PRIDE [34] partner repository with the data set identifier PXD027278 [35].

## Abbreviations

*B. inositovora*: *Babjeviella inositovora*
*C. albicans*: *Candida albicans*
*C. lusitaniae*: *Clavispora lusitaniae*
*C. tropicalis*: *Candida tropicalis*
*E. coli*: *Escherichia coli*
FDR: False discovery rate
LC-MS/MS: Liquid chromatography-tandem mass spectrometry
LeuRS: leucine-tRNA synthetase
*M. acaciae*: *Millerozyma acaciae*
PSM: Peptide spectrum match
