# Supplementary material for: Proteogenomics analysis of CUG codon translation in the human pathogen Candida albicans
Source: BMC Biol. 2021 Dec 4;19:258. doi: 10.1186/s12915-021-01197-9 (PMC8645108; doi:10.1186/s12915-021-01197-9)
Supplement: Supplementary file 1 — Additional file 1 : Table S1. MS/MS data processing statistics. [file 12915_2021_1197_MOESM1_ESM.pdf]

Table S1. MS/MS data processing statistics.

	**Total**				**CTG codon**
	# PSM	Mass error [ppm] Mean	# identified proteins	% identified proteins	# CUG pos covered where CUG pos is part of a chain of supported pos
*C. albicans*_SC5314_1	272308	0,612	2683	21.77	232
*C. albicans*_SC5314_2	128043	0,628	3682	29.88	386
*C. albicans*_SC5314_3	378920	0,617	4361	35.39	682
*C. albicans*_SC5314_4	246148	0,633	3849	31.23	447
*C. albicans*_SC5314_5	253059	0,613	4064	32.98	582
*C. albicans*_SC5314_6	294823	0,603	4035	32.74	568
*C. albicans*_SC5314_7	280560	0,622	4047	32.84	574
*C. albicans*_SC5314_8	234502	0,603	3730	30.27	369
*C. albicans*_SC5314_9	235716	0,610	3948	32.04	455
*C. albicans*_WO1_1	259622	0,605	2377	39.66	212
*C. albicans*_WO1_2	118851	0,617	3096	51.66	344
*C. albi*cans_WO1_3	351209	0,605	3586	59.84	606
*C. albicans*_WO1_4	233439	0,623	3228	53.86	415
*C. albicans*_WO1_5	240531	0,598	3394	56.63	546
*C. albicans*_WO1_6	279849	0,590	3367	56.18	532
*C. albicans*_WO1_7	266590	0,611	3386	56.50	537
*C. albicans*_WO1_8	222209	0,589	3123	52.11	340
*C. albicans*_WO1_9	223937	0,596	3304	55.13	431
C. dubliniensis	97318	0,650	2136	36.03	110
C. tropicalis	292120	0,607	2587	41.34	241
M. acaciae	157601	0,290	3390	71.26	1000
B. inositovora	223556	0,670	3439	54.39	766
C. lusitaniae	125927	0,643	3571	60.16	1513
