# Supplementary material for: Proteogenomics analysis of CUG codon translation in the human pathogen Candida albicans
Source: BMC Biol. 2021 Dec 4;19:258. doi: 10.1186/s12915-021-01197-9 (PMC8645108; doi:10.1186/s12915-021-01197-9)
Supplement: Supplementary file 3 — Additional file 3 : Tables S2-S4. Table S2: Gene and codon numbers in the annotation of the diploid Candida albicans strain SC5314. Table S3: CTG, CTT and TCC codons and the corresponding codons in the other allele in the annotation of the diploid Candida albicans strain SC5314. Table S4: Identification of peptides with codons corresponding to CTG or TCC in the other allele in the annotation of the diploid Candida albicans strain SC5314. [file 12915_2021_1197_MOESM3_ESM.pdf]

Table S2. Gene and codon numbers in the annotation of the diploid *Candida albicans* strain SC5314.

	total	CTG	CTT	TCC
genes in gene prediction	12421			
genes not tagged with A/B	20			
genes tagged with A/B	12303			
gene + other allele	6119			
gene + other allele, same length	5804			
gene + other allele, completely identical	2172			
gene + other allele, identical at codon		1303	1846	1851
gene + other allele, differing at codon		215	430	759
codon pos, other codon in other allele		244	576	1000

Table S3. CTG, CTT and TCC codons and the corresponding codons in the other allele in the annotation of the diploid *Candida albicans* strain SC5314. Differences at the first, second and third codon position are highlighted in blue, green and orange, respectively.

other codon	CTG	CTT	TCC			CTG	other aa	CTT	other aa	TCC	other aa
TTT	4	100	3		all	244		576		1000	
TTC	1	3	66		pos-1	61	L M V	178	F I V	95	P A T
TTA	1	7	3		%	25,0%		30,9%		9,5%	
TTG	42	2	1		pos-2	84	P Q R	75	P H R	73	F Y C
CTT	5		3		%	34,4%		13,0%		7,3%	
CTC	7	178	1		pos-3	58	L	234	L S	761	S
CTA	46	51			%	23,8%		40,6%		76,1%	
CTG		5			rest	41		89		71	
ATT	3	44			%	16,8%		15,5%		7,1%	
ATC		1	1								
ATA	2	3	2								
ATG	8										
GTT	1	34	1								
GTC		2									
GTA		1	1								
GTG	11										
TCT	1	4	523								
TCC		3									
TCA	2	4	165								
TCG	1		73								
AGT		2									
AGC			2								
CCT	1	54	1								
CCC		2	46								
CCA		3									
CCG	48										
ACT	1	1	5								
ACC		1	28								
ACA	1										
ACG	1		1								
TAT	1	1									
TAC		1	11								
GCT		3									
GCC		1	21								
GCA		2									
GCG	1										
CAT		13	2								
CAC			2								
CAA	4	4									
CAG	30	1									
AAT		9	9								
AAC		1	3								
AAA		8	8								
AAG	1		1								
GAT	2	3	6								
GAC											
GAA	3	3	2								
GAG		2									
TGT	1										
TGC	4		6								
TGG	1	4									
CGT	1	8									
CGC											
CGA	1	1									
CGG	6										
AGA		2	1								
AGG			1								
GGT		4									
GGC											
GGA	1		1								
GGG											

Table S4. Identification of peptides with codons corresponding to CTG or TCC in the other allele in the annotation of the diploid *Candida albicans* strain SC5314. In case of CTG, the peptides with CTG (= serince) have not been found but only peptides of the other allele. In case of CTT, peptides of the corresponding allele have not been found. In case of TCC, in cannot be revealed to which allele the peptide belongs.

	CTG codon			TCC codon	
gene and codon pos (only supported pos)	g11596-23 (CTG/CAG)	g3472-295 (CTG/TTG)	g4531-417 (CTG/CAG)	TCA/TCC/TCG	g27-103 (TCC/AGC);
*C. albicans*_SC5314_1		1	1		
*C. albicans*_SC5314_2				24 [a]	
*C. albicans*_SC5314_3				35 [b]	1
*C. albicans*_SC5314_4	1			25 [c]	1
*C. albicans*_SC5314_5		1		29 [d]	1
*C. albicans*_SC5314_6		1		29 [e]	1
*C. albicans*_SC5314_7		1		26 [f]	1
*C. albicans*_SC5314_8		1		21 [g]	1
*C. albicans*_SC5314_9		1		26 [h]	1

[a] g331-165 (TCC/TCT); g421-219 (TCC/TCA); g483-71 (TCC/TCT); g1085-179 (TCC/TCT); g1573-60 (TCC/TCT); g1781-114 (TCC/TCT); g1781-116 (TCC/TCT); g1808-385 (TCC/TCA); g2364-211 (TCC/TCT); g2765-71 (TCC/TCG); g2862-247 (TCC/TCT); g4008-978 (TCC/TCT); g4782-327 (TCC/TCT); g5893-22 (TCC/TCT); g6351-94 (TCC/TCT); g7585-1656 (TCC/TCT); g7699-666 (TCC/TCT); g7996-345 (TCC/TCG); g8811-84 (TCC/TCA); g9097-252 (TCC/TCT); g11209-329 (TCC/TCT); g11311-245 (TCC/TCT); g11713-89 (TCC/TCT); g12263-353 (TCC/TCT);

[b] g17-531 (TCC/TCT); g27-103 (TCC/AGC); g421-219 (TCC/TCA); g636-30 (TCC/TCT); g948-636 (TCC/TCA); g1074-136 (TCC/TCT); g1085-179 (TCC/TCT); g1573-60 (TCC/TCT); g1781-114 (TCC/TCT); g1781-116 (TCC/TCT); g1808-385 (TCC/TCA); g2025-26 (TCC/TCG); g2364-211 (TCC/TCT); g2765-71 (TCC/TCG); g2862-247 (TCC/TCT); g6351-94 (TCC/TCT); g11798-272 (TCC/TCA); g3575-15 (TCC/TCT); g12263-353 (TCC/TCT); g8711-487 (TCC/TCT); g12259-109 (TCC/TCT); g4008-978 (TCC/TCT); g4274-399 (TCC/TCT); g11799-409 (TCC/TCT); g11311-245 (TCC/TCT); g11713-89 (TCC/TCT); g4782-327 (TCC/TCT); g5671-61 (TCC/TCT); g11209-329 (TCC/TCT); g7585-1656 (TCC/TCT); g7699-666 (TCC/TCT); g7996-345 (TCC/TCG); g8811-84 (TCC/TCA); g9031-67 (TCC/TCT); g9097-252 (TCC/TCT);

[c] g27-103 (TCC/AGC); g421-219 (TCC/TCA); g3206-757 (TCC/TCA); g636-30 (TCC/TCT); g1573-60 (TCC/TCT); g1781-114 (TCC/TCT); g1781-116 (TCC/TCT); g1808-385 (TCC/TCA); g5644-502 (TCC/TCT); g1925-295 (TCC/TCT); g2364-211 (TCC/TCT); g7720-98 (TCC/TTC); g2862-247 (TCC/TCT); g6351-94 (TCC/TCT); g12263-353 (TCC/TCT); g12259-109 (TCC/TCT); g3822-54 (TCC/TCT); g11713-89 (TCC/TCT); g4782-327 (TCC/TCT); g11209-329 (TCC/TCT); g7585-1656 (TCC/TCT); g7699-666 (TCC/TCT); g7996-345 (TCC/TCG); g8811-84 (TCC/TCA); g9097-252 (TCC/TCT);

[d] g17-531 (TCC/TCT); g27-103 (TCC/AGC); g421-219 (TCC/TCA); g636-30 (TCC/TCT); g6503-32 (TCC/TCT); g1074-136 (TCC/TCT); g1085-179 (TCC/TCT); g1216-812 (TCC/TCT); g1573-60 (TCC/TCT); g1808-385 (TCC/TCA); g5644-502 (TCC/TCT); g1925-295 (TCC/TCT); g2364-211 (TCC/TCT); g7720-98 (TCC/TTC); g2862-247 (TCC/TCT); g6351-94 (TCC/TCT); g11798-272 (TCC/TCA); g3575-15 (TCC/TCT); g12263-353 (TCC/TCT); g4274-399 (TCC/TCT); g4782-327 (TCC/TCT); g5671-61 (TCC/TCT); g6992-631 (TCC/TCT); g11209-329 (TCC/TCT); g7585-1656 (TCC/TCT); g7699-666 (TCC/TCT); g7996-345 (TCC/TCG); g8811-84 (TCC/TCA); g9097-252 (TCC/TCT);

[e] g27-103 (TCC/AGC); g421-219 (TCC/TCA); g1074-136 (TCC/TCT); g1085-179 (TCC/TCT); g11422-76 (TCC/TCT); g1781-114 (TCC/TCT); g1781-116 (TCC/TCT); g1808-385 (TCC/TCA); g5644-502 (TCC/TCT); g1925-295 (TCC/TCT); g2364-211 (TCC/TCT); g2862-247 (TCC/TCT); g3916-193 (TCC/TCT); g3916-206 (TCC/TCT); g6351-94 (TCC/TCT); g11798-272 (TCC/TCA); g3575-15 (TCC/TCT); g12263-353 (TCC/TCT); g3822-54 (TCC/TCT); g4274-399 (TCC/TCT); g11799-409 (TCC/TCT); g11311-245 (TCC/TCT); g11713-89 (TCC/TCT); g4782-327 (TCC/TCT); g5671-61 (TCC/TCT); g11209-329 (TCC/TCT); g7585-1656 (TCC/TCT); g7996-345 (TCC/TCG); g9097-252 (TCC/TCT);

[f] g27-103 (TCC/AGC); g421-219 (TCC/TCA); g6503-32 (TCC/TCT); g1074-136 (TCC/TCT); g1201-404 (TCC/TCT); g1573-60 (TCC/TCT); g1781-114 (TCC/TCT); g1781-116 (TCC/TCT); g1808-385 (TCC/TCA); g1925-295 (TCC/TCT); g2025-26 (TCC/TCG); g2364-211 (TCC/TCT); g2862-247 (TCC/TCT); g3916-206 (TCC/TCT); g6351-94 (TCC/TCT); g11798-272 (TCC/TCA); g3575-15 (TCC/TCT); g12263-353 (TCC/TCT);

g4274-399 (TCC/TCT); g11799-409 (TCC/TCT); g11311-245 (TCC/TCT); g4782-327 (TCC/TCT); g11209-329 (TCC/TCT); g7585-1656 (TCC/TCT); g7996-345 (TCC/TCG); g9097-252 (TCC/TCT);

[g] g27-103 (TCC/AGC); g421-219 (TCC/TCA); g1085-179 (TCC/TCT); g1781-114 (TCC/TCT); g1781-116 (TCC/TCT); g1808-385 (TCC/TCA); g5644-502 (TCC/TCT); g1925-295 (TCC/TCT); g2364-211 (TCC/TCT); g2862-247 (TCC/TCT); g6351-94 (TCC/TCT); g11798-272 (TCC/TCA); g12263-353 (TCC/TCT); g12259-109 (TCC/TCT); g11311-245 (TCC/TCT); g4782-327 (TCC/TCT); g6992-631 (TCC/TCT); g11209-329 (TCC/TCT); g7585-1656 (TCC/TCT); g7996-345 (TCC/TCG); g9097-252 (TCC/TCT);

[h] g27-103 (TCC/AGC); g421-219 (TCC/TCA); g636-30 (TCC/TCT); g948-636 (TCC/TCA); g1074-136 (TCC/TCT); g1085-179 (TCC/TCT); g1573-60 (TCC/TCT); g1781-114 (TCC/TCT); g1781-116 (TCC/TCT); g1808-385 (TCC/TCA); g5644-502 (TCC/TCT); g2364-211 (TCC/TCT); g2862-247 (TCC/TCT); g6351-94 (TCC/TCT); g11798-272 (TCC/TCA); g3575-15 (TCC/TCT); g8711-487 (TCC/TCT); g12259-109 (TCC/TCT); g4274-399 (TCC/TCT); g11311-245 (TCC/TCT); g4782-327 (TCC/TCT); g6992-631 (TCC/TCT); g11209-329 (TCC/TCT); g7585-1656 (TCC/TCT); g7996-345 (TCC/TCG); g9097-252 (TCC/TCT);
