# Supplementary material for: Proteogenomics analysis of CUG codon translation in the human pathogen Candida albicans
Source: BMC Biol. 2021 Dec 4;19:258. doi: 10.1186/s12915-021-01197-9 (PMC8645108; doi:10.1186/s12915-021-01197-9)
Supplement: Supplementary file 5 — Additional file 5 : Tables S5-S7. Table S5: MS/MS data processing statistics against CTT and TCC databases. Table S6: Number of obtained PSMs and proteins and CTT positions covered. Table S7: Number of obtained PSMs and proteins and TCC positions covered. [file 12915_2021_1197_MOESM5_ESM.pdf]

Table S5. MS/MS data processing statistics against CTT and TCC databases.

	# PSM	# non-redundant peptides	Mass error [ppm] Mean	# identified proteins	% identified proteins
*C. albicans*_WO1_2_CTT	354035	29742	0.592	3282	54.76
*C. albicans*_WO1_3_CTT	327291	33818	0.603	3532	58.94
*C. albicans*_WO1_4_CTT	219365	25899	0.621	3151	52.58
*C. albicans*_WO1_5_CTT	223557	31138	0.597	3331	55.58
*C. albicans*_WO1_6_CTT	195210	23629	0.585	3153	52.61
*C. albicans*_WO1_7_CTT	247844	31265	0.609	3324	55.46
*C. albicans*_WO1_8_CTT	205545	26412	0.586	3026	50.49
*C. albicans*_WO1_9_CTT	206727	29204	0.592	3221	53.75
*C. albicans*_WO1_2_TCC	355136	30198	0.609	3341	55.75
*C. albicans*_WO1_3_TCC	326667	34430	0.614	3576	59.67
*C. albicans*_WO1_4_TCC	218535	26284	0.631	3193	53.28
*C. albicans*_WO1_5_TCC	223308	31552	0.610	3369	56.22
*C. albicans*_WO1_6_TCC	260238	31581	0.600	3335	55.65
*C. albicans*_WO1_7_TCC	247637	31564	0.621	3359	56.05
*C. albicans*_WO1_8_TCC	204269	26551	0.597	3057	51.01
*C. albicans*_WO1_9_TCC	206337	29505	0.605	3250	54.23

Table S6. Number of obtained PSMs and proteins and CTT positions covered. If not indicated otherwise, numbers include positions both with and without direct b-/y-type support.

	# PSM covering CTT	# supported PSMs covering CTT	# CTT pos covered	# supported CTT pos covered	% supported pos	# identified proteins with CTT	% identified proteins with CTT	CTT recovery [%]
*C. albicans*_WO1_2_CTT	27670	19976	2727	2135	78.29	2793	52.2	18.97
*C. albicans*_WO1_3_CTT	28859	20987	3374	2641	78.28	3061	57.2	20.03
*C. albicans*_WO1_4_CTT	18655	13036	2520	1920	76.19	2690	50.27	18.01
*C. albicans*_WO1_5_CTT	19359	14229	2991	2380	79.57	2860	53.45	19.09
*C. albicans*_WO1_6_CTT	16863	12103	2261	1752	77.49	2702	50.5	15.83
*C. albicans*_WO1_7_CTT	21352	15167	3024	2311	76.42	2860	53.45	19.34
*C. albicans*_WO1_8_CTT	17278	11597	2507	1829	72.96	2587	48.35	18.38
*C. albicans*_WO1_9_CTT	18353	12411	2862	2105	73.55	2796	52.25	18.48

Table S7. Number of obtained PSMs and proteins and TCC positions covered. If not indicated otherwise, numbers include positions both with and without direct b-/y-type support.

	# PSM covering TCC	# supported PSMs covering TCC	# TCC pos covered	# supported TCC pos covered	% supported pos	# identified proteins with TCC	% identified proteins with TCC	TCC recovery [%]
*C. albicans*_WO1_2_TCC	63750	39653	3187	2378	74.62	2933	55.67	23.13
*C. albicans*_WO1_3_TCC	58163	35757	3657	2784	76.13	3160	59.97	23.82
*C. albicans*_WO1_4_TCC	39831	23591	2945	2172	73.75	2814	53.41	21.91
*C. albicans*_WO1_5_TCC	39895	25177	3398	2607	76.72	2978	56.52	23.51
*C. albicans*_WO1_6_TCC	45991	28409	3425	2541	74.19	2950	55.99	24.27
*C. albicans*_WO1_7_TCC	43535	26241	3396	2497	73.53	2975	56.46	23.47
*C. albicans*_WO1_8_TCC	36692	20135	2965	2039	68.77	2719	51.6	22.84
*C. albicans*_WO1_9_TCC	36507	20278	3220	2277	70.71	2899	55.02	22.51
